# WMH and AD: is CAA the missing piece? Potential consequences at the anti‐amyloid era

**DOI:** 10.1002/alz.089566

**Published:** 2025-01-09

**Authors:** Andreas Charidimou, Department of Neurology, Boston University School of Medicine, Boston, MA, USA

**Affiliations:** ^1^ VA Boston Healthcare System, Boston, MA USA

## Abstract

Underlying cerebral amyloid angiopathy (CAA) is extremely common in patients with Alzheimer’s disease, contributing to cognitive impairment and hampering anti‐amyloid treatments safety. Distinct patterns of white matter hyperintensities (WMH) are supporting markers of CAA and are now included in the Boston criteria, along other hemorrhagic features of the disease. These WMH patterns includes a multispot WMH pattern in the cortical‐subcortical regions and posterior predominance of WMH. This presentation will focus on THE core imaging markers of CAA, including clinical interpretation of WMH in the context of suspected CAA, associated features and potential relevance for ARIA in anti‐amyloid treatments.

